# MicroRNA-29 induces cellular senescence in aging muscle through multiple signaling pathways

**DOI:** 10.18632/aging.100643

**Published:** 2014-03-12

**Authors:** Zhaoyong Hu, Janet D. Klein, William E. Mitch, Liping Zhang, Ivan Martinez, Xiaonan H. Wang

**Affiliations:** ^1^ Renal Division, Department of Medicine, Emory University, Atlanta, GA 30322, USA; ^2^ Nephrology, Department of Medicine, Baylor College of Medicine, Houston, TX 77030, USA; ^3^ Immunology and Cell Biology, Department of Microbiology, West Virginia University, Morgantown, WV 26506, USA

**Keywords:** p50, p16Ink4A, RB, B-myb, sarcopenia, p85, IGF-1

## Abstract

The mechanisms underlying the development of aging-induced muscle atrophy are unclear. By microRNA array and individual qPCR analyses, we found significant up-regulation of miR-29 in muscles of aged rodents vs. results in young. With aging, p85α, IGF-1 and B-myb muscle levels were lower while the expression of certain cell arrest proteins (p53, p16 and pRB) increased. When miR-29 was expressed in muscle progenitor cells (MPC), their proliferation was impaired while SA-βgal expression increased signifying the development of senescence. Impaired MPC proliferation resulted from interactions between miR-29 and the 3'-UTR of p85a, IGF-1 and B-myb, suppressing the translation of these mediators of myoblast proliferation. *In vivo*, electroporation of miR-29 into muscles of young mice suppressed the proliferation and increased levels of cellular arrest proteins, recapitulating aging-induced responses in muscle. A potential stimulus of miR-29 expression is Wnt-3a since we found that exogenous Wnt-3a stimulated miR-29 expression 2.7-fold in primary cultures of MPCs. Thus, aging-induced muscle senescence results from activation of miR-29 by Wnt-3a leading to suppressed expression of several signaling proteins (p85α, IGF-1 and B-myb) that act coordinately to impair the proliferation of MPCs contributing to muscle atrophy. The increase in miR-29 provides a potential mechanism for aging-induced sarcopenia.

## INTRODUCTION

The loss of muscle mass in older subjects, termed sarcopenia, not only decreases muscle strength, contributing to a high incidence of accidental falls and injuries but also compromising the quality of life of elderly subjects [1]. Identification of mechanisms and contributors to aging-induced muscle loss could lead to new therapeutic strategies for preventing and treating sarcopenia. Thus, we examined mechanisms causing sarcopenia and the development of muscle cell senescence in both mice and rats.

Cellular processes that have been proposed to explain aging-induced sarcopenia include changes in hormone levels [2], inflammation-initiated impairment in protein synthesis and acceleration of protein degradation in muscle [3], responses to accumulated reactive oxygen species [4] and alterations in factors that regulate myogenesis [5]. We found evidence that cellular sense-cence also contributes to the processes of the sarcopenia of aging. This is relevant because cellular senescence leads to irreversible growth arrest from defective processes of cellular repair [6] and chronic inflammation [3]. This response can impact tumor suppression (growth arrest in early life) and tumor promotion (chronic inflammatory response in late life) [7].

Certain proteins have been identified as markers of cellular senescence and they interfere with the processes of proliferation of progenitor cells. Specific markers that have been identified include p16^Ink4A^, retinoblastoma protein tumor suppressor (RB), protein 53 (p53) and SA-βgal. For example, p16^Ink4A^ (multiple tumor suppressor 1) was identified as a marker of senescence because it inhibits the cell cycle kinase, Cdk4 while activating RB [8, 9]. Alternatively, an increase in p53 is associated with inhibition of cellular proliferation in muscles of aging rodents and hence, p53 has been identified as a marker of senescence [10]. A widely used marker of senescence in both cells and tissue is senescence-associated β-galactosidase (SA-βgal) [11]. Although SA-βgal is not required for senescence, when it is increased, it serves as the most extensively used biomarker of senescent cells. Moreover, it is a reliable marker of senescence and is easy to detect *in situ* and *in vitro*.

Another candidate marker of senescence includes microRNAs because they can interfere with cell growth signaling pathways. However, it is not clear whether a specific microRNA can mediate cellular senescence [12]. Examining this possibility is complex because it is established that a single microRNA can influence the translation of more than one individual mRNA while multiple microRNAs can influence the translation of a single mRNA. For example, a microRNA identified as a regulator of p53 expression could be considered a senescence marker but there are reports that p53 can be regulated by microRNA-20 (miR-20) [13], miR-106a [14], miR-22 [15], miR-33 [16] and miR-29 [17]. To determine if a microRNA can regulate the expression of senescence processes, we studied miR-29 because we and others find that miR-29 is increased in aging skeletal muscle [18, 19]. In addition, miR-29 can cause apoptosis of cholangiocarcinoma and hepatocellular carcinoma cells [20, 21]. miR-29 also can influence senescence processes: in HeLa/E6 cells, miR-29 was up-regulated during progression towards cellular senescence [17]. In addition, it was shown that miR-29 can target the 3'-UTR of the mRNA of B-myb (myeloblastosis-related protein B). This is relevant because B-myb is a transcription factor that can regulate the expression of genes that augment cell proliferation, suppress tumorgenesis and inhibit cellular senescence [22]. In the HeLa/E6 cells, miR-29-induced inhibition of B-myb would block the inhibitor of senescence resulting in the development of cellular senescence [17]. Whether B-myb causes the same responses in aged muscle and suppresses cellular senescence is largely unknown.

Aging is associated with a decrease in the IGF-PI3K-Akt signaling pathway [23]. This is relevant because IGF-1 plays a critical role in myogenesis by stimulating both myoblast proliferation and differentiation to restore the proliferation of satellite cells in immobilized skeletal muscles of aged mice [24]. A decrease in IGF-1 resulting from miR-29 targeting of IGF-1 and the p85α regulatory subunit of phosphatidylinositide 3-kinase (PI3K) could cause an important senescence response in muscle. Notably, miR-29 has two binding sites on the 3'-UTR of IGF-1 and it has been determined in a murine osteoblastic cell line, MC3T3-E1, that miR-29 directly targets the 3'-UTR of IGF-1 mRNA during osteoblast differentiation [25]. There also is a binding site for miR-29 on the 3'-UTR of p85α. Park et al. found that in HeLa cells, miR-29 directly binds to the mRNA of p85α, suppressing p85α protein expression [26].

Are there other pathways that initiate aging-induced muscle cell senescence? In mesenchymal stem cells, activation of Wnt signaling leads to cellular senescence by a pathway that includes activated p53 plus DNA damage responses [27]. In addition, prolonged exposure to Wnt-3a triggers cellular senescence *in vitro* and *in vivo* in various cells (e.g., fibroblasts [28], hematopoietic stem cells [29, 30], human mammary artery cells [31] and thymocyte cells [32] . The response was not limited to cultured cells but was also demonstrated in an accelerated aging mouse model [33]. In skeletal muscle, Wnt-induced responses differ during myogenesis *vs.* aging: during embryogenesis Wnt-3a signaling is required for muscle formation and in young mice, Wnt-3a stimulates differentiation of myogenic cells to promote growth [34]. In aging muscle, however, activation of the canonical Wnt signaling pathway converts satellite cells from a myogenic to a fibrogenic lineage. This lineage conversion can be suppressed by wnt inhibitors [35].

To determine the impact of miR-29 on muscle metabolism and function in aging, we examined whether miR-29 influences the development of senescence in muscles of aging rodents. We extended the results to determine if miR-29 acts through regulatory proteins such as IGF-1, p85, and B-myb. Finally, we evaluated how Wnt-3a influences miR-29 promoter activity to produce the muscle sarcopenia of aging.

## RESULTS

### miR-29 levels and skeletal muscle mass in aged rodents (Table 1)

**Table 1:** Muscle Weight and content

	units	4 month (n=5)	28 month (n=5)	P value
EDL weight	mg	179.4 ± 11.1	151.1 ± 8.9	0.041
Soleus weight	mg	169.2 ± 9.5	147.3 ± 6.5	0.042
EDL muscle mass index*	g muscle/g b.w.	43.2 ± 2.6	36.3 ± 1.3	0.007
Soleus muscle mass index*	g muscle/g b.w.	42.8 ± 3.5	37.1 ± 1.8	0.015

All data presents as mean ± s.e.; P<0.05 is significant (aging *vs.* young), n=6.

* muscle mass index = (muscle weight / body weight) x100.

In Fisher 344 rats, we compared results from “young” (4 months of age) to those of “old” (28 months of age) rats. Muscle weights corrected for body weights are shown in Table 1: weights of both EDL (predominantly glycolytic, fast-twitch, white fiber) and soleus muscles (predominantly oxidative, slow-twitch, red fiber) were significantly decreased in old *vs.* young rats. We previously published that similar reduced muscle weights were found in C57BL6 mice [36]. Thus, aging induces sarcopenia in both types of muscle fibers.

**Figure 1:**
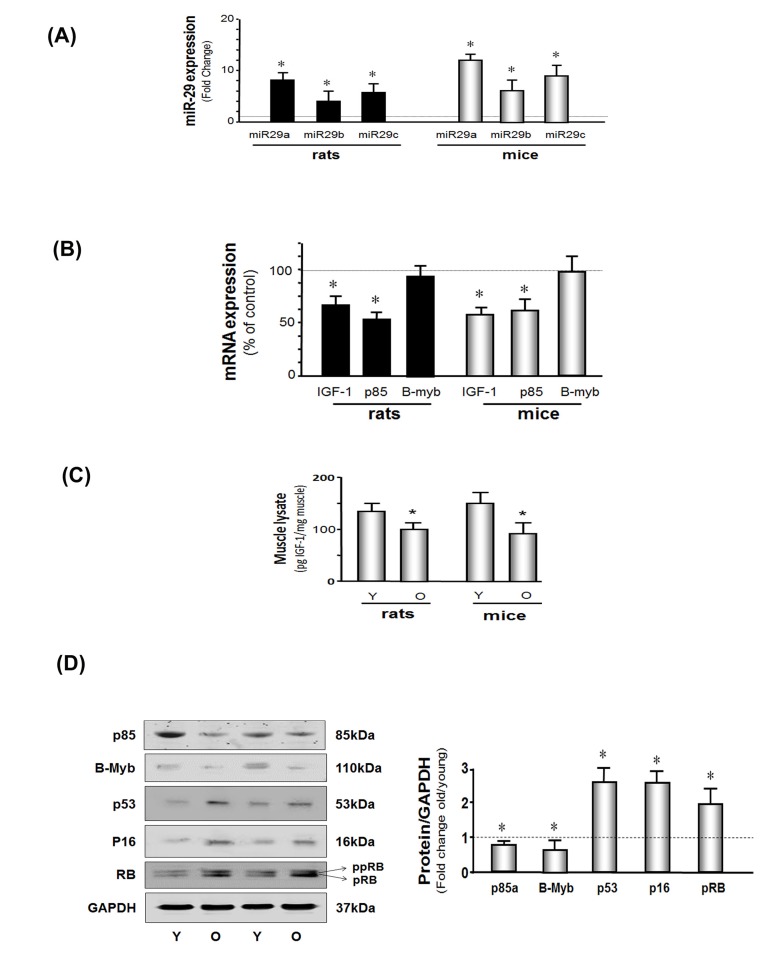
miR-29 and cellular arrest proteins are increased and IGF-1, p85 and B-myb are decreased in the muscles of aged rodents (**A**) Total RNA obtained from hind-limb muscles of young and aged rats and mice were assayed for miR-29a, b and c by real time qPCR. The bar graph shows miR-29a, b and c in aged rodents muscles expressed as a fold change above the control (young rodent) which is represented by a line at 1-fold. Results are normalized to U6 RNA (Bars: mean ± s.e.; n=6 pairs; *p<0.05 *vs.* young). (**B**) Total RNA from hind-limb muscles of young and aged rats and mice were assayed for IGF-1, p85 and B-myb expression by real time qPCR. The bar graph shows IGF-1, p85 and B-myb in aged rodents muscles expressed as a percentage of control (young rodent) which is represented by a line at 100%. Results are normalized to 18S RNA (Bars: mean ± s.e.; n=6 pairs; *p<0.05 *vs.* young). (**C**) IGF-1 protein level was measured by ELISA in muscle lysates from young and old rodents. Results in the bar graph compare the amount of IGF-1 in muscle from old (O) *vs.* young (Y) rats and mice. All data were normalized to the muscle total protein concentration (Bars: mean ± s.e.; n=6; *p<0.05 vs. young). (**D**) p85α B-myb, p53, p16^INK4A^, RB and GAPDH proteins were measured by western blotting of muscle lysates from young and old mice. Two bands of the RB protein were detected: the lower band is hypophosphoryated RB (pRB; MW 107 kDa) while the upper band is more highly phosphorylated RB protein (ppRB; MW 112kDa). Results in the bar graph compare the densities of protein bands in aged muscle expressed as a fold-change from levels in young mice which is represented by a line at 1-fold. All band densities were normalized to the density of GAPDH (Bars: mean ± s.e.; n=6; *p<0.05 *vs.* young).

Next, we analyzed results of microRNA microarrays from muscles of young (4 months of age) or old (28 months of age) 344 Fisher rats. By parametric analysis, 41 microRNAs were significantly different in muscles of old *vs*. young rats (Supplement 2). However, when the analysis was based on the more rigorous, volcano plot-Benjamin analysis, 21 microRNAs were significantly different in muscles of old *vs*. young rats (Table 2). Two of the 21 microRNAs, were substantially increased in muscles of old rats *vs.* results in muscles from young rats. With aging, mir-29a increased 13-fold while miR-29c was 5-fold higher in results compared to results in muscles from young rats. By the parametric analysis miR-29b was also significantly increased (Supplement 2) but not when results were analyzed by the volcano plot-Benjamin method. We also measured miR-29a, b, and c using real-time qPCR (Figure 1A). In muscles of aged *vs.* young rats, miR-29a, b and c were increased 10-, 7- and 8-fold, respectively (P<0.05). Likewise, miR29a-c levels in muscles of aged mice were significantly greater (12- 9- and 11-fold, respectively) compared to results from muscles of young mice. Thus, miR-29 subtypes in muscle are up-regulated by aging in both mice and rats.

**Table 2:** RNA microarray analyses of muscles of young and aged rats

Column	Detector	DDCt Old-Young	Log10RQ Old-Young	Fold	Inverted fold <1
117	**hsa-miR-29c-4373289**	-3.7197	1.11974	**13.1747**	
115	**hsa-miR-29a-4373065**	-2.34506	0.705935	**5.0808**	
249	mmu-miR-376c-4378112	1.900019	−0.57196	0.2679	−3.732
372	rno-miR-450-4381124	2.063852	−0.62128	0.2392	−4.181
263	mmu-miR-451-4373360	2.34447	−0.70576	0.1969	−5.079
228	mmu-miR-322-4373332	2.362548	−0.7112	0.1944	−5.143
244	mmu-miR-369-5p-4378118	2.660478	−0.80088	0.1582	−6.322
32	hsa-miR-134-4373141	2.820302	−0.849	0.1416	−7.063
275	mmu-miR-483-4381035	3.071588	−0.92464	0.1189	−8.407
240	mmu-miR-351-4373345	3.097419	−0.93242	0.1168	−8.559
136	hsa-miR-335-4373045	3.351132	−1.00879	0.0980	−10.204
247	mmu-miR-376a-4373347	3.605993	−1.08551	0.0821	−12.176
293	mmu-miR-543-4378111	3.78297	−1.13879	0.0726	−13.765
150	hsa-miR-379-4373023	4.034841	−1.21461	0.0610	−16.391
158	hsa-miR-433-4373205	4.422924	−1.33143	0.0466	−21.450
250	mmu-miR-379-4373349	5.136886	−1.54636	0.0284	−35.185
170	hsa-miR-539-4378103	5.457241	−1.64279	0.0228	−43.933
276	mmu-miR-485-3p-4386764	5.979852	−1.80011	0.0158	−63.112
259	mmu-miR-434-3p-4373358	6.175273	−1.85894	0.0138	−72.267
277	mmu-miR-487b-4378116	6.270174	−1.88751	0.0130	−77.181
24	hsa-miR-127-4373147	6.948719	−2.09177	0.0081	−123.530

### In muscles of aged mice, the protein levels of IGF-1, p85α and b-myB are low while cell cycle arrest proteins are increased

A microRNA targeting data base search (http://www.targetscan.org) revealed that miR-29a, b and c have multiple target sites (e.g., there are at least 855, highly conserved sites in 760 mRNAs). We chose to study IGF-1, p85α and b-myB because we and others have shown that activation of the IGF-PI3K (p85α)-Akt intracellular signaling pathway improves muscle protein metabolism and myogenesis [37]. In addition, an increase in B-myb promotes the expression of genes involved in cell proliferation and the limitation of senescence [22, 38]. Thus, we measured IGF-1, p85α and B-myb in muscles of aged and young mice. Aging muscle was associated with a decrease in the mRNAs encoding IGF-1 and p85α, but not B-myb (Figure 1B) while the protein levels of IGF-1, p85α, and B-myb were decreased in muscles of aged mice (Figure 1C & D). Since the effect of microRNA is to either inhibition of translation (with no change on mRNA amount) or de- gradation mRNA (with decreasing mRNA). A decrease in the protein may be the result of decreased mRNA, inhibited translation (e.g. B-myb) or both. Aging was also associated with a general increase in cell arrest proteins (Figure 1D). Muscle levels of the tumor inhibitor protein, p53, were 2.7-fold higher and the cell cycle arrest protein, p16^Ink4A^,was 2.6-fold higher in aged mice (P<0.05). Finally, the active, hypophosphorylated form of RB (pRB: active form) and the inactive, more highly phosphorylated form off RB (ppRB) were increased to a comparable degree (2.6- and 2.1-fold respectively). The proliferation marker protein, Ki67, was decreased in aged muscle vs. young (Supplement 3), which indicates MPCs proliferation is decreased in aging mice.

### In primary cultured MPCs, miR-29 decreases myo-blast proliferation and induces cellular senescence

**Figure 2:**
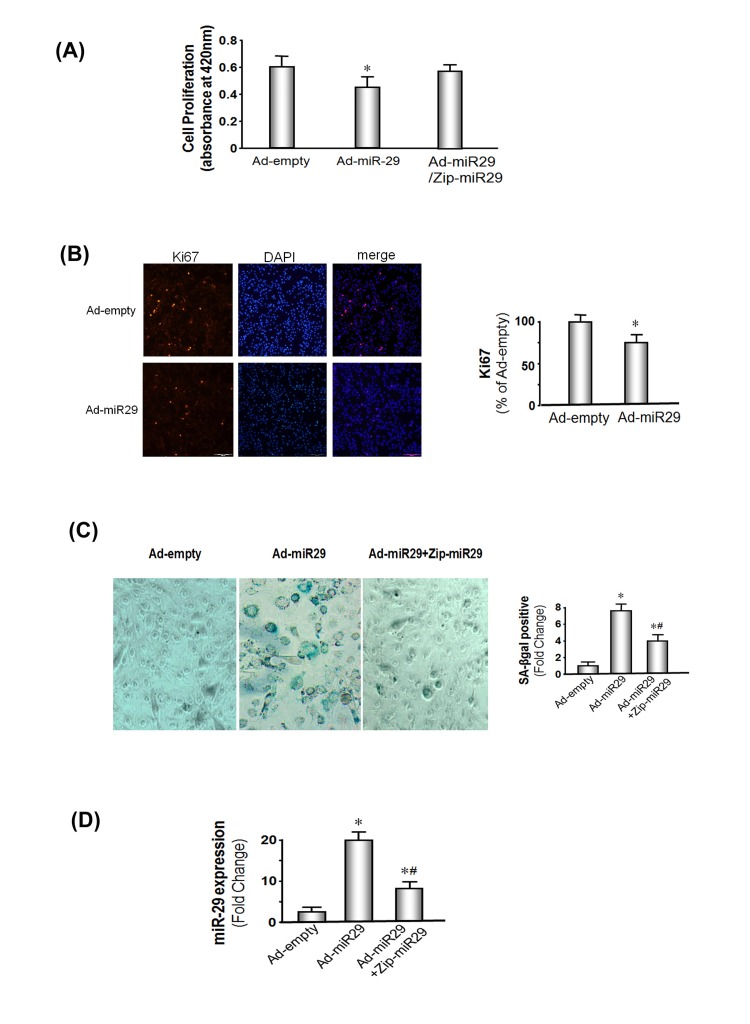
miR-29 expression decreases muscle cell proliferation and induces cellular senescence in MPCs (**A**) MPCs were transduced with Ad-miR-29 or the control adenovirus (Ad-empty). Proliferation was assessed using a chromogenic substrate and a commercial kit (Millipore) monitoring absorbance at 420 nm (Methods). The bars show the mean ± s.e. (n=6; *p<0.05 *vs.* Ad-empty). (**B**) MPCs were transduced with Ad-miR-29 or Ad-empty. The Ki67 expression, a marker of proliferation, was assessed by immunohistology. A positive Ki67 was determined as a bright red spot localized in the nuclei. The bar graph shows cells that were positively stained for Ki67 in miR-29 treated cells and are expressed as a percent of the positive cells in the Ad-empty treated cells. Counts were made in 5 pre-defined, randomly chosen fields; each field had >500 nuclei. Red staining in nuclei was assessed using the Micro-suite Five Biological Software (Bars: mean ± s.e.; n=6/condition; *p<0.05 *vs.* ad-empty). (**C**) MPCs were transduced with Ad-miR-29 or with the Ad-empty adenovirus. To block miR-29 expression in MPCs, vectors that express an antisense of Zip-miR29 (pmiRZip29a plus pmiRZip29c) were transfected into MPCs 4 hours *before* the Ad-miR-29 or Ad-empty viruses were added. The blue color identifies SA-βgal is present in cells with the “fried egg morphology” that signifies cellular senescence. The bar graph shows the percentage of cells with positive staining of SA-βgal in 5 pre-defined, randomly chosen fields (Bars: mean ± s.e.; n=6/condition; *p<0.05 *vs.* controls; #p<0.05 *vs.* Ad-miR-29). (**D**) MPCs were transduced with Ad-miR-29 or with Ad-empty adenovirus. To block miR-29 expression in MPCs, vectors that express an antisense of miR-29a+c (pmiRZip29a plus pmiRZip29c) were transfected into MPCs 4 hours *before* the Ad-miR29 or Ad-empty (Ctrl) viruses were added. miR-29a expression was measured using real-time qPCR; U6 was the internal control. The bar graph shows the amount of miR-29a expressed as a fold change from the level in controls (Bars: mean ± s.e.; ctrl defined as 1 fold; n=3 determinations per condition; *p<0.05 and #p<0.01 vs. Ad-miR29 only).

To study the impact of miR-29 on muscle cell growth, MPCs were transduced with the Ad-miR-29 adenovirus or Ad-empty (control) and cells cultured in normal growth medium. Cell proliferation was assessed using a cell proliferation assay kit (Millipore, Burlington, MA). The proliferation rate was 28% decreased in MPCs cultured with miR-29 overexpressing cells compared to proliferation in cells treated with Ad-empty (P<0.05; Figure 2A). The results were confirmed by measuring the proliferation marker protein, Ki67. There was a 25% suppression of Ki67 in MPCs expression of exogenous miR-29 (Figure 2B). These data indicate miR-29 decreases MPCs proliferation.

Next, we examined the influence of longer term expression of miR-29 by transducing MPCs with the Ad-miR-29 adenovirus. The expression was boosted by adding fresh Ad-miR-29 or Ad-empty when the media was changed every 2 days and at each passage. 1.5X10^6^ cells were seeded in 100 mm dishes and passaged before becoming confluent. We found that after 3 passages, increased miR-29 expression led to the development of flat cells resembling the “fried eggs” pattern that is indicative of cell senescence (Supplement 4). In addition, miR-29 increases muscle cell senescence was supported by the finding that the senescence marker, SA-βgal. In MPCs that were transduced by the Ad-empty adenovirus SA-βgal was almost undetectable. In contrast, SA-βgal positive cells were sharply increased following transduction with Ad-miR-29 (Figure 2C). In addition, expression of zip-miR-29 (an inhibitor of miR-29) significantly lowered miR-29 expression levels by about 60% (Figure 2D), and led to a 55% decrease in SA-βgal in zip-miR-29 containing-MPCs compared with miR-29 only (Figure 2C). Similar to the responses in muscles of aged mice, treatment of MPCs with Ad-miR-29 decreased the expressions of B-myb and p85α but increased the expression of the cellular arrest proteins, p53, p16 and pRB (Supplement 5).

### miR-29 expression in muscle increases cell cycle arrest proteins and cellular senescence

**Figure 3:**
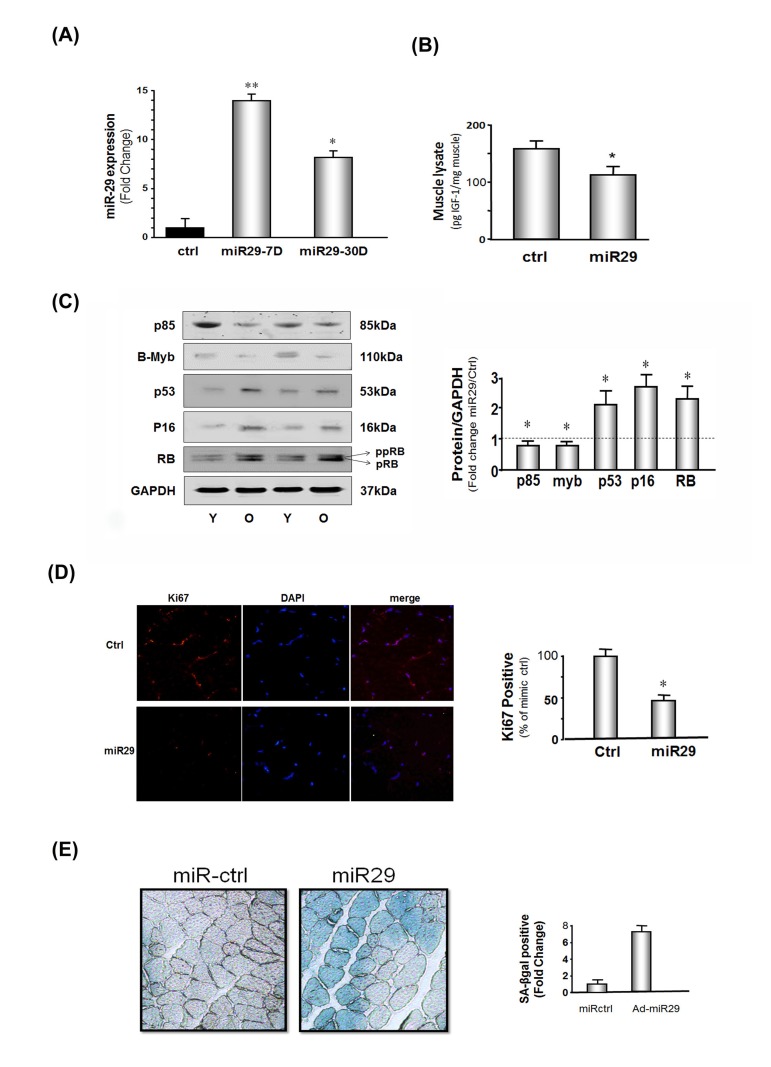
miR-29 expression in muscles of mice decreases IGF-1, p85α, B-myb but increases cell cycle arrest proteins The mouse microRNA mimic, miR-29a (mmu-miR-29a), was injected into the right tibialis anterior (TA) muscle of 4 month old normal mice; permeation increased by muscle electroporation. The left TA muscle was used as a control and injected with mouse mimic miR-ctrl (mmu-miR-ctrl) and electroporated. Muscles were harvested at 7 and 30 days after electroporation. (**A**) RNA isolated from TA muscles and miR-29a expressions were measured using real-time qPCR; U6 was the internal control. The bar graph shows the amount of miR-29a expressed as a fold change from the level in controls (Bars: mean ± s.e.; ctrl defined as 1 fold; n=3 determinations per condition; *p<0.05 and **p<0.01 *vs.* control). (**B**) IGF-1 protein level was measured by ELISA in muscle lysates. Results in the bar graph compare the protein amount of IGF-1 in control muscle (ctrl) *vs.* miR-29 overexpressing muscle. All data were normalized to the muscle total protein concentration (Bars: mean ± s.e.; n=6; *p<0.05 vs. control). (**C**) Protein levels of P85α, B-myb, P53, P16^INK4A^, RB and GAPDH in lysates from muscles at 7 days after electroporation were measured by Western blotting. There were two RB protein bands: the lower is hypophosphoryated RB (pRB; MW 107 kDa) and the upper band is more highly phosphorylated RB protein (ppRB; MW 112kDa). The bar graph shows the density of each protein band expressed as a fold-change from control levels (mimic miR-ctrl was set to 1, indicated by a horizontal line in the graph). All band densities were normalized to the density of GAPDH (Bars: mean ± s.e.; n=6; *p<0.05 *vs.* ctrl). (**D**) The proliferation marker (Ki67) was assessed by immuno-histochemistry in TA muscles at 30 days after electroporation of mmu-miR-29 or mmu-miR-ctrl into muscles. The bar graph shows the percentage of positive staining nuclei of Ki67 in 10 predetermined, random fields for each condition(Bars: mean ± s.e.; n=6/condition; *p<0.05 *vs.* controls). (**E**) The level of the senescence marker, SA-βgal, was assessed in cross sections of TA muscles at 30 days after electroporation of the mmu-miR-29 or mu-miR-ctrl. The blue color shows the presence of SA-βgal indicating the presence of cellular senescence. The bar graph shows the percentage of positive staining in 10 predetermined, random fields for each condition(Bars: mean ± s.e.; n=6/condition; *p<0.05 *vs.* controls).

To determine if these miR-29-induced results in cultured cells occur *in vivo*, we electroporated tibialis anterior (TA) muscles of young mice with a miR-29 mimic, mouse miR-29 (mmu-miR-29a). After 7 days, miR-29 expression was increased 12-fold and after 30 days, it was 8-fold higher (Figure 3A). On day 7 following the electroporation, IGf-1, p85α and B-myb were decreased in the muscles of mice that were over-expressing miR-29 (Figure 3B & C). Thus, exogenous miR-29 not only suppresses components of the IGF-1/Akt pathway but also led to a 2.1-fold increase in p53 and a comparable increase in two other cell cycle arrest proteins, p16^Ink4A^ and pRB (Figure 3C). Notably, these results are consistent with our finding of the levels of the same proteins in muscles of old vs. young mice (Figure 1C & D). This response was confirmed by finding a decreased level of the proliferation marker, Ki67 in muscle (Figure 3D). Finally, evidence that these responses reflect the development of senescence, we found that the increase in the muscle level of miR-29 at 30 days after electroporation of mmu-miR-29 and this accompanied by an increase in the level of the senescence marker, SA-β-gal (Figure 3E).

### miR-29 binds to the 3'-UTR of IGF-1, p85α and B-myb

**Figure 4:**
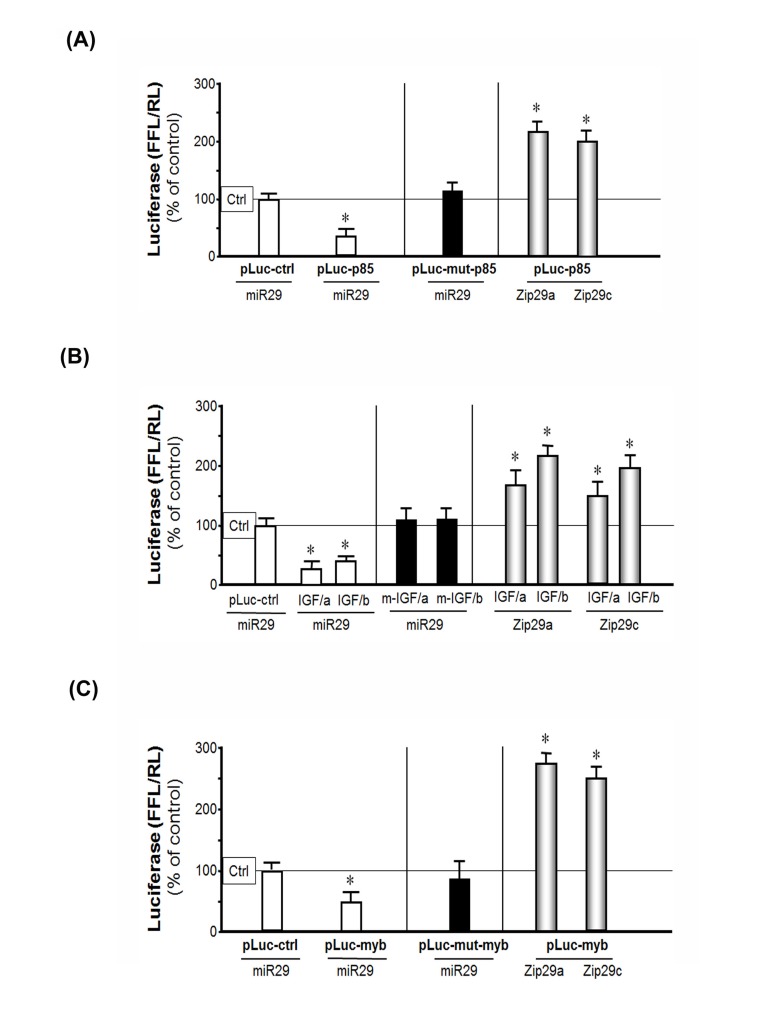
miR-29 binds to the 3'-UTR of p85α, IGF-1 and B-myb in MPCs suppressing their translation (luciferase activity) (**A**) MPCs were transfected with pLuc-ctrl, pLuc-p85α-3'-UTR or pLuc-mutant-p85α (p85α with a mutated 3'-UTR binding site for miR-29). Then they were treated with the empty virus (Ctrl), the miR-29 adenovirus to over-express miR-29 (miR-29) or miR-29 inhibitor (Zip29a or Zip29c). Luciferase activity in cells that received the control virus was designated as the 100% activity level (designated by a horizontal line in the graph). The other bars all show the response of the cells to miR-29 or zip-miR-29 (indicated below each bar) expressed as a percent of the control level for each experiment. Left (white bars): the effect of miR-29 on p85α 3'-UTR; middle (black bar): the effect of miR-29 on mutated p85α 3'-UTR; right (gray bars): the effect of inhibited endogenous miR-29 on p85α 3'-UTR. Triplicate determinations were made in each condition and each experiment was repeated a total of three times; the results were combined to calculate differences in firefly luciferase activity normalized by renilla luciferase activity. The data represent mean ± s.e.; (n=9; *p<0.05 *vs.* Ad-ctrl).(**B**) MPCs were transfected with constructs containing the two miR-29 binding sites on IGF-1 3'-UTR, pMIR-IGF/321-3217 (IGF/a) and pMIR-IGF/3275-5574 (IGF/b), before being treated with the empty adenovirus (Ctrl), Ad-miR-29 (miR29) or miR-29 inhibitor (Zip29a or Zip29c). Luciferase activity in cells that received the control virus was designated as the 100% activity level (designated by a horizontal line in the graph). The other bars all show the response of the cells to miR-29 or zip-miR-29 (indicated below each bar) expressed as a percent of the control level for each experiment. Left (white bars): the effect of miR-29 on the two IGF 3'-UTR binding sites (IGF/a or IGF/b); middle (black bars): the effect of miR-29 on mutated (m-) IGF/a or IGF/b; right (gray bars): inhibited endogenous miR-29 effect on IGF/a or IGF/b. The results were combined to calculate differences in firefly luciferase activity normalized by renilla luciferase activity. The data represent mean ± s.e.; (n=9; *p<0.05 *vs.* Ad-ctrl). (**C**) MPCs were transfected with pLuc-ctrl, pLuc-3'UTR-B-myb (pLuc-myb) or pLuc-mutant-B-myb (pLuc-mut-myb). Then they were treated with the empty virus (ctrl), the miR-29 adenovirus (miR29) to over-express miR-29 or miR-29 inhibitor (Zip29a or Zip29c). Luciferase activity in cells that received the control virus was designated as the 100% activity level (designated by a horizontal line in the graph). The other bars all show the response of the cells to miR-29 or miR-29 inhibitor (Zip29a or Zip29c) expressed as a percent of the control level for each experiment. Left (white bars): the effect of miR-29 on B-myb 3'-UTR; middle (black bar): the effect of miR-29 on mutated B-myb 3'-UTR; right (gray bars): inhibited miR-29 effect on B-myb 3'-UTR. The results were combined to calculate differences in firefly luciferase activity normalized by renilla luciferase activity. The data represent mean ± s.e.; (n=9; *p<0.05 *vs.* Ad-ctrl).

To determine if expression of the IGF-1, p85α and B-myb are affected by miR-29, we examined changes in three firefly luciferase constructs, each containing the 3'-UTR mRNA sequence of IGF-1 or p85α or B-myb. Each of the constructs was transfected separately into MPCs and luciferase activity responding to miR-29 was measured. First, we found that the expression of miR-29 (induced by Ad-miR-29) was associated with a 66% decrease in p85α (pLuc-3UTR-p85) luciferase activity compared to results in cells treated with the Ad-empty virus (Figure 4A- white bar). These results indicate that miR-29 specifically interacts with the 3'-UTR of p85α and decreases its ability to produce p85α protein. This inhibition response was abolished when miR-29 binding site was mutated, since this mutation (pLuc-3URT_mutant_-p85) prevents miR-29 binding to targets in the mRNA (Figure 4A- black bar). The specificity of this result was confirmed by inhibition of endogenous miR-29 by antisense vectors of miR-29a (Zip-miR29a) or mir-29C (Zip-miR29C). The presence of the antisense produced a 2-fold increase in p85α luciferase activity (Figure 4A- gray bar). These results demonstrate that an inhibition of endogenous miR-29 increases the luciferase activity of p85α.

Second, we tested whether miR-29 will target IGF-1 in MPCs. The miR-29 has 2 target sites in the 3'-UTR of the IGF-1 mRNA. The first is located at 962-969 nt and the second at 3593-3600 nt of 3'-UTR of IGF-1. MPCs were transfected with two vectors, each designed to incorporate different binding sites: pMIR-IGF/321-3217 (IGF/a: containing the first target site) and pMIR-IGF/3275-5545 (IGF/b: containing the second target). When MPCs were transfected with these vectors, transduction with miR-29 caused an 86% decrease in target 1 luciferase activity; and a 61% decrease in luciferase in cells treated with the second target site *vs.* results in MPCs that had been treated with control virus. Mutation of either of the two binding sites on the 3'-UTR of IGF-1 diminished the miR-29 activation. Inhibition of endogenous miR-29 by antisense vectors Zip-miR29a or Zip-miR29c produced the increasing in IGF-1 luciferase activity (Figure 4B). Third, transduction of MPCs with exogenous miR-29 also decreased B-myb luciferase activity compared with the results in cells treated with the Ad-empty adenovirus. This response was abolished when the miR-29 binding site was mutated. Inhibition of miR-29 with the antisense vectors led to a 2.7-fold increase in luciferase activity (Figure 4C). Thus, miR-29 specifically interacts with the 3'-UTR of IGF-1, P85α and B-myb to decrease their expressions.

### miR-29 promoter activity was up-regulated by Wnt-3a in MPCs

To determine what might impact the expression of miR-29 in muscle, we assayed mir-29 promoter activity. miR-29 has three subtypes, miR29a, b and c. These subtypes are located at different position on the chromosomes. miR-29a and miR-29b1 are clustered on human chromosome 7 and mouse chromosome 6. miR-29c and miR-29b2 are clustered on human and mouse chromosome 1. We used two luciferase reporter construct: PGL3-miR-29a/b1 (DNA sequence: -1530 to +165) and pGL3-miR-29c/b2 (DNA sequence: -4500 to +3) to test miR-29 promoter activity.

**Figure 5:**
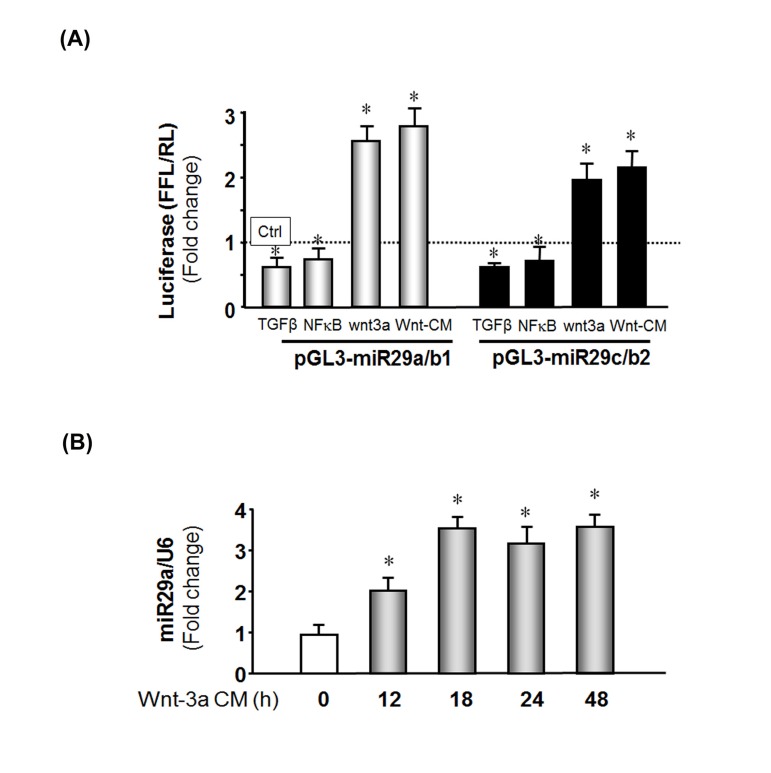
Wnt-3a induces miR-29 promoter activation (**A**) MPCs were transfected with pGL3-miR-29a/b1 (gray bar) or pGL3-miR-29C/b2 (black bar) to assay miR-29 promoter activities. Transfected cells were treated as follows: left to right, treated with TGF-β; co-transfected with NFκB plasmid (pCMV1.p65); transduced with Ad-Wnt-3a; or treated with Wnt-3a-conditioned media. The bar graph represents firefly luciferase activity corrected for renilla luciferase activity (FFL/RL) and compared to control (luciferase activity of pGL3 untreated, set to 1 and designated by a horizontal line in the graph). The data represent the means ± s.e.; (n=9, *p<0.05 *vs.* pGL3 (Ctrl). (**B**) MPCs were cultured in Wnt-3a conditioned media and cells harvested at the indicated times. Total RNA was isolated and miR-29a expression was measured by qPCR. The bar graph shows miR-29a from Wnt-3a conditional medium expressed as a fold-change *vs.* miR-29a levels in cells cultured with control media (set to 1). Results are normalized to U6 RNA as an internal control. The data represent the means ± s.e.; (n=3 pairs; *p<0.05 *vs.* control).

First, we investigated two candidate proteins that have been reported to regulate miR-29 expression in other systems; TGF-β [39] and NF-κB [40]. We exposed MPCs to TGF-β or we express NF-κB by transfecting NF-κB p65 subunit into the cells and found 28% (by NF-κB) or 33% (by TGF-β) decrease in miR-29 promoter activity (Figure 5A). The magnitude of these responses is similar to that in earlier reports [39, 40]. There was, however, a consistent increase in miR-29 in aging muscle *vs.* young muscle. Thus, TGF-β and NF-κB are not responsible for miR-29 changes in aging muscle senescence.

In previous work, we and other found that circulating Wnt-3a, increases with aging [33, 35, 36]. We examined whether Wnt-3a in aging muscles could contribute to the changes in proteins that influence the cell cycle. We overexpressed Wnt-3a in MPCs using a recombinant adenovirus (Ad-Wnt-3a) or added Wnt-3a conditioned media (Methods) to determine if these would activate miR-29 promoter. As shown in Figure 5A, MPCs that were transduced with Ad-wnt-3a exhibited a 2.5-fold increase in miR-29a/b1 and a 1.9-fold increase in miR-29c/b2 promoter activities. The miR-29 promoter activities were also increased by 2.8-fold in miR-29a/b1 and a 2.3-fold in miR-29c/b2 promoter when MPC cultured in wnt-3a conditional media. Finally, we examined whether Wnt-3a interacts with miR-29 by treating MPCs with Wnt-3a conditioned media and measured miR-29a, b and c expression by qPCR. Wnt-3a treatment increased miR-29a in time-dependent manner (Figure 5B) as were miR-29b and miR-29c (data not shown). These observations indicate that Wnt-3a up-regulates miR-29 promoter activity and increase miR-29 production, but does not provide information about direst binding of wnt-3a to the miR-29 promoter [41].

## DISCUSSION

To evaluate potential molecular mechanisms that cause aging-induced sarcopenia, we studied aged rats and mice and found that the microRNA, miR-29 can initiate muscle cell senescence leading to aging-induced sarcopenia. miR-29 was increased in muscles of both mice and rats and it was associated with the presence of higher levels of cellular arrest proteins and lower levels of cell proliferation. Expression of miR-29 suppressed the expression of IGF-1 and p85α and B-myb and led to induction of senescence *in vivo*. Thus, the presence of senescent cells that are derived from muscle progenitor cells would contribute to an exhaustion of MPCs regeneration, contributing to the development of muscle atrophy and sarcopenia.

Does miR-29 change in aging senescence? miR-29 is up-regulated in the liver and muscles of Zmpste24-/- (Hutchinson-Gilford progeria syndrome) mice, a murine model of aging [18]. In addition, miR-29 is down-regulated in a model of delayed onset of aging, the Ames dwarf mouse [42]. miR-29 was also found to be increased during spontaneous senescence in fibroblasts [17]. A recent study concluded that in the aged brain miR-29 was increased and correlates with the reduction of insulin-like growth factor-1 [19] In our study, we found that miR-29 were increased in aged muscle of rodents and related with sarcopenia.

The next question is how does the increase in miR-29 that occurs in aging impair cell proliferation? Our results indicate that the increase in miR-29 suppresses IGF-1, p85α or B-myb, resulting in decreased translation of these mediators of cell proliferation and muscle growth. The inhibition of IGF-1, p85α or B-myb provides a mechanism that is consistent with the ability of an increase in IGF-1 to promote satellite cell proliferation. In fact, there is evidence that IGF-1 can restore the suppressing of satellite cell proliferation that occurs with aging [24]. An allied mechanism could involve administration of Mechano-Growth Factor (MGF), an isoform of IGF-1: when primary muscle cell cultures isolated from neonatal or young adults are exposed to MGF, there is an increase in the proliferative potential and a delay in the senescence of satellite cells. In contrast, MGF does not exert this response in primary cultures of muscle cells from aged adults [43].

The cellular arrest proteins, p16^Ink4A^ and p53 promote cellular senescence [10, 44]. The mechanism underlying this association could involve a decrease in the transcription factor, B-myb [45, 46]. Results from human embryonic lung fibroblast cells indicate that B-myb can down-regulate the cellular arrest protein, p16^Ink4A^, by inhibiting its transcription. Knockdown of B-myb was shown to up-regulate p16^Ink4A^ [45] and B-myb can suppress the influence of p53 [46]. Our finding that an increase in miR-29 in muscles from aged rodents results in decreased levels of B-myb provides an explanation for the senescence occurring when there is up-regulation of p53 and RB (Figure 6).

How does aging impair MPCs proliferation? We and others have found that miR-29 stimulates myoblast differentiation into myotubes [47, 48]. However, in aging muscle, an increase in miR-29 results in inhibition of proliferation of muscle progenitor cells (Figure 2B). In addition, we find that miR-29 is maintained at a high level in muscles of aging rodents. Associated with this finding, there is inhibition of muscle cell proliferation with loss of myogenesis from muscle progenitor cells. Thus, muscle cells become senescent and are incapable of contributing to muscle growth. In agreement with this formulation, an elegant study by Marzi et al. found that 35 miRNAs were induced during myoblast differentiation (named differentiation-associated miRNA). Seven of the differentiation-associated miRNAs acted as negative regulators of proliferation [49]. Notably, miR-29 was among the negative regulators of proliferation. Other recent information indicates that miR-29 can target Akt3 resulting in reduced proliferation with facilitated differentiation of myoblasts into skeletal muscle [50]. That is, in aging muscle, miR-29 is constantly at a high level, so proliferation is decreased leading to lower MPC levels. Even though miR-29 increases differentiation, cell base for this differentiation is inadequate leading to muscle senescence.

How are miR-29 levels increased in muscle of aging rodents? There are conflicting views concerning the regulation of miR-29 promoter activity. Some investigators report that aging increases miR-29 in liver and muscle [17, 18, 42]. In mouse fibroblasts, Ugalde et al. concluded that aging caused DNA damage which activates miR-29a/b1 and miR-29c/b2 promoter activity [18]. Others report that c-Myc, Hedgehog and NF-κB transcription factors suppress miR-29 a/b1 promoter activity in cholangiocarcinoma cells [40]. In addition, TGF-β reportedly down-regulates miR-29 transcription via an NF-κB pathway. This would suggest there should be a lower level of miR-29 in muscle and possibly other tissues [51] but we and others have found that miR-29 is consistently up-regulated at least in muscle of aging rodents. We have no explanation why there reports are conflicting.

To understand the regulation of miR-29 transcription in myoblasts, we investigated Wnt signaling. Along with miR-29, Wnt-3a is essential for myogenic differentiation. Wnt signaling appears to trigger the switch from proliferation of MPCs to differentiation in adult skeletal muscle. Previously, we found that Wnt-3a is increased in aging muscle [36]. In the current study we show that Wnt-3a stimulates the promoter activity of miR-29. We interpret this as an indirect upreglation of miR-29 expression, since Wnt-3a is a signaling protein rather than a transcription factor [52]. There are reports which support this finding: first, Brack et al. report that Wnt-3a is significantly increased in muscles of aged rodents [35]. Second, Kapinas et al. found that Wnt signaling will induce miR-29a transcription activation via an interaction between two TCF/LEF1 (T-cell factor/lymphoid enhancer factor-1) transcription factors which can bind to sites in the miR-29 promoter [41].

In conclusion, we have uncovered 4 lines of evidence that miR-29 activation determines cellular senescence in muscle: 1) an increase in miR-29 significantly up-regulates the senescence marker, SA-βgal, *in vivo and in vitro*; 2) an increase in miR-29 stimulates the expression of mediators of cell growth arrest, p53, p16^Ink4A^ and pRB, *in vivo* and *in vitro; 3)* an increase in miR-29 suppresses the expression of mediators of cell proliferation, IGF-1, P85α and B-myb; 4) expression of miR-29 in MPCs significantly suppresses their proliferation *in vivo* and *in vitro.* The results identify a novel pathway that contributes to the aging-induced loss of muscle mass and senescence. The pathway proceeds from activation of Wnt-3 to increase miR-29 expression which acts in a coordinated way to suppress the expression of proteins regulating muscle growth, IGF-1, p85, B-myb and possibly other factors. In addition, the increases in miR-29 upregulates the expressions of cell arrest proteins, p53 and p16^Ink4A^ in muscle. These events impair proliferation of muscle progenitor cells and contribute to the development of muscle cell senescence and loss of muscle mass.

## EXPERIMENTAL PROCEDURES

### MicroRNA array and quantitative real-time polymerase chain reaction (qPCR)

The RNA population enriched in small RNAs was extracted from muscles using the mirVana miRNA isolation kit (Ambion, Austin, TX). MicroRNA profiling was performed using the Rodents miRNA Taqman Low Density Array (v1.0; containing 368 miRNAs; Applied Biosystems, Foster City, CA). RNA sample preparation, hybridization, post-hybridization processing and image scanning were performed by the Virology & Drug Discovery Core of the Emory Center for AIDS Research. Individual microRNAs were measured by real time qPCR as described [53]. Reverse transcription of 20 ng of RNA samples was performed using a miRCUTY LNA Universal cDNA synthesis kit (Exiqon INC., Woburn, MA); real-time qPCR was assayed with the miRCUTY LNA microRNA PCR SYBR Green master mix (Exiqon INC).

### Luciferase reporter assay and transfection

For transfection, cells in growth media were seeded in 12-well plates and transfected using Effectene transfection reagent (Qiagen, Valencia, CA). In each well, 0.3 μg of firefly luciferase vector and 0.08 μg of the *Renilla* luciferase (control vector) were introduced. After 48 hours, firefly and *Renilla* luciferase activities were measured by dual-luciferase assays (Promega) using TD-20/20 Luminometer (Turner designs, Sunnyvale, CA) [54]. We calculated the results as the ratio of firefly luciferase to renilla luciferase (x100). The lentivector-based anti-microRNA expression vectors, pmiRZip29a, and b, were purchased from SBI system Biosciences (Mountain View, CA), and used to inhibit of miR-29a or/and b activation in MPCs. TGF-β was purchased from Sigma-Aldrich (St. Louis, MO, USA) and the pCMV4.p65 (NF-κB p65 subunit) plasmid was from Addgene (Cambridge, MA, USA). For the promoter construct of miR-29a/b1, the DNA sequence (-1530 to +165) flanking miR-29 transcriptional start site (+1) was ligated into the pGL3 reporter [40]. To construct the miR-29c/b2 Luc reporter plasmid, a ~ 4.5 kb fragment of transcriptional site was cloned into the Xho I and Hind III sites of the pGL3-basic vector [48].

### Muscle progenitor cell (MPC) isolation

MPC were isolated from hind-limb muscles of 4 month old mice as described (36, 54). Briefly, muscles were minced into a coarse slurry and gently agitated for 1 h at 37°C in DMEM with 25 mM HEPES (pH 7.4) plus 0.1 % pronase (Calbiochem, San Diego, California). The digest was passed through a 100-μm filter and then centrifuged through a percoll gradient (70% overlaid with 40%) (36). Cells from the gradient interface were collected and cultured on collagen-coated dishes. To remove fibroblast contamination, isolated MPCs were pre-plated to uncoated dishes and incubated at 5% CO2, 37°C for 30 minutes. The supernatant fractions containing the cells that had not adhered were again exposed to uncoated dishes again. Finally the supernatant was transferred to a collagen coated dish. This procedure was repeated at the beginning of each passage. The MPCS were cultured in Ham's F-10 Nutrient Mixture medium (Invitrogen) with 20% fetal bovine serum, 100 u/ml penicillin, 100 μg/ml streptomycin. Maintenance media for MPCs contained 5 ng/ml human β-fibroblast growth factor (Atlanta Biologicals, Atlanta, GA), but this was removed before the experiments. Immunohistology was used to assess the degree of culture purity. MPCs were identified using anti-myoD antibodies. Anti-α-smooth muscle actin (Sigma) was used to identify fibroblast contamination. Cells collected were >90% MyoD positive and <10% α-smooth muscle actin positive (supplement 1).

### Wnt-3a conditioned media

Was produced by culturing L-wnt3a cells (ATCC: CCL-2647) which secrete Wnt3a. Conditioned media from the parental cell line (ATCC CRL-2648) was used as control media. Basal media for both cell lines was DMEM/F12 with 10% fetal bovine serum, 100 U/ml penicillin and 100 μg/ml streptomycin. Maintenance media for L-wnt3a cells contained 0.4 mg/ml G-418; it was removed before the experiments. Cells were grown in 50% Wnt-3a conditioned medium (or control media) plus 50% normal growth media.

### Animal

Fisher 344 rats were purchased from Charles River (Wilmington, MA) and mice (C57BL/6J) from Jackson Laboratories (Bar harbor, ME). All experiments involving animals were approved by the Institutional Animal Care and Use Committee of Emory University. For electroporation, the mimic mmu-miR-29 was injected into the tibialis anterior (TA) muscle [55]. A pair of electrode needles (fixed at 7mm distance) was inserted into the muscle to a depth of 5 mm to bracket the injection site and pulses were delivered using an Electro Square Porator (T820, BXT, San Diego, CA). Optimal conditions were 5 pulses of 85 volt, 50 μsec per pulse, 100 μsec duration.

### Immunoblotting and Antibodies

To identify skeletal muscle proteins, hind-limb muscles were homogenized in RIPA buffer and proteins in the soluble fraction were assessed by Western blot as described (55). Primary antibodies were: B-myb (R&D System, Minneapolis, MN); p53, and p16Ink4A (Santa Cruz Biotechnology, Santa Cruz, CA); myoD and myogenin (DSHB product, Iowa, IA), RB (BD Pharmingen, San Jose, CA), Ki-67 and GAPDH (Millipore Burlington, MA), and p85α of PI3K (Upstate Biotechnology, Temecula, CA).

### Cell proliferation assay

A cell proliferation assay kit (Millipore, Burlington, MA) was used according to manufacturer's instructions. Briefly, MPCs (1x104/well in a 96-well microplate) were cultured in normal growth media. After 24 hours, 10 μL WST-1/ECS chromogenic solution was added to each well, and incubated at 37oC for an additional 4 hours. The absorbance of the samples at 420 nm was determined using a TECAN microtiter plate reader with Magellan software (Morrisville, NC, USA).

### Virus Reagents

All experiments involving recombinant virus were approved by the Environmental health and safety Office of Emory University. Ad-miR-29a/b/c was produced by Dr. Fude Fang (Peking Union Medical College) and Ad-wnt-3a were produced by Dr. Tong-Chuan He (Chicago University (56, 57); they were amplified as described (57). The adenovirus titer (transduction unit, TU) was determined by visually assessing fluorescent cells that had been transduced by serial dilutions of the adenoviruses. For viral transduction, cultured cells were sub-cultured in growth media on day 1, and 5 μl of the concentrated viral preparation (109 TU) was added to 2-ml media and applied to 6-well plates; > 90% of cells were transduced (based on expression of the green fluorescent protein).

### Statistical Analysis

All data are reported as the mean ± the standard error of the mean. Comparison between groups was performed with the Kruskal-Wallis one-way analysis of variance, with p<0.05 being considered significant. For comparisons between 2 groups, a Student's T test was used and p<0.05 being considered significant. The microRNA array data were analyzed by GeneSpring GX 9.0 (Mathworks, Natick, MA) with a GC robust multi-array analysis (GC-RMA) followed by a Volcano-Benjamin analysis to identify the most strikingly different results.

## SUPPLEMENTAL FIGURES



## Abbreviations

miR-29: microRNA 29
RB: Retinoblastoma protein
SA-βgal: senescence-associated β-galactosidase
p16Ink4A: inhibitor of the proliferative kinase Cdk4 protein 16
