# Supplementary material for: MicroRNA-29 induces cellular senescence in aging muscle through multiple signaling pathways
Source: Aging (Albany NY). 2014 Mar 12;6(3):160–75. doi: 10.18632/aging.100643 (PMC4012934; doi:10.18632/aging.100643)
Supplement: Supplementary file 1 [file aging-06-160-s001.pdf]

SUPPLEMENTAL DATA

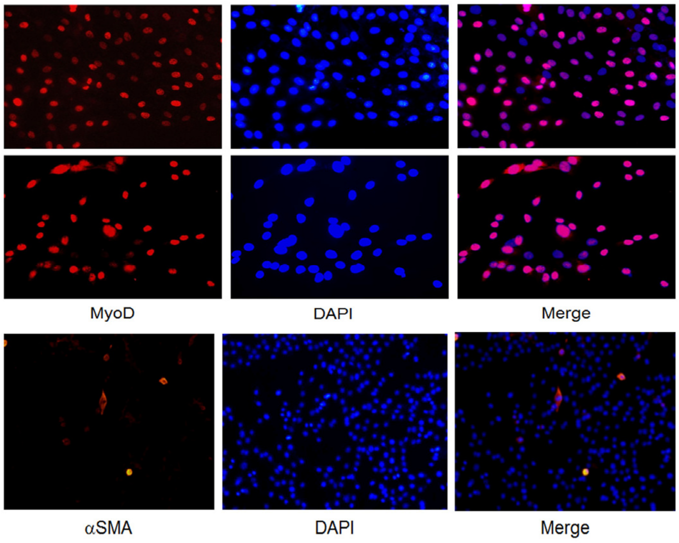

Supplement 1. Primary cultured MPCs. (**A**) The muoD expression (a marker of myogenesis MPCs) was assessed in the first of third passaged cells by immunohistology. A positive myoD was determined as a red spot localized in the nuclei. (**B**) α-smooth muscle actin (a marker of myofibroblast was assessed by immunohistology in third passaged of MPCs. The positive α-smooth muscle actin was stained red color.

hsa-miR-136-4373173	4.483898	2.36E-03	2.36E-03	-0.74533	6.989839	15.68084	16.72015	11.83812	11.59507	flagged	valid	target_not_	-1.34979	Significant
hsa-miR-184-4373113	9.474225	1.57E-02	1.57E-02	-2.54417	4.08006	23.43819	17.11872	11.09119	10.51727	flagged	valid	target_not_	-2.85203	Significant
hsa-miR-299-5p-4373188	14.32767	3.85E-05	3.85E-05	1.520634	20.50139	23.43819	24.64041	9.984597	9.438666	flagged	valid	target_not_	-4.31306	Significant
hsa-miR-32-4373056	6.834766	4.79E-02	4.79E-02	-3.71543	2.838431	23.43819	17.39548	12.26478	14.89936	flagged	valid	target_not_	-2.05747	Significant
hsa-miR-380-5p-4373021	13.21327	5.15E-05	5.15E-05	1.437747	19.02412	23.43819	24.64041	10.5756	11.07647	flagged	valid	target_not_	-3.97759	Significant
hsa-miR-382-4373019	17.94763	1.38E-05	1.38E-05	1.745449	26.66927	23.43819	24.64041	6.160588	6.022761	flagged	valid	target_not_	-5.40277	Significant
hsa-miR-455-4378098	12.40177	6.96E-05	6.96E-05	1.3417	17.60674	23.43819	24.64041	11.33802	11.93704	flagged	valid	target_not_	-3.73331	Significant
mmu-miR-337-4373338	14.79882	3.87E-03	3.87E-03	-1.18107	6.103885	18.00107	24.64041	6.495013	6.548839	flagged	valid	target_not_	-4.45489	Significant
mmu-miR-434-5p-4373359	13.97555	4.05E-05	4.05E-05	1.507083	20.24021	23.43819	24.64041	9.836493	10.29101	flagged	valid	target_not_	-4.20706	Significant
mmu-miR-495-4381078	15.07045	1.27E-05	1.27E-05	1.759248	27.22354	23.43819	22.87787	8.087202	8.087968	flagged	valid	target_not_	-4.53666	Significant
mmu-miR-540-4378119	12.84937	2.34E-04	2.34E-04	0.83336	12.87277	23.43819	24.64041	12.22536	10.15451	flagged	valid	target_not_	-3.86805	Significant
mmu-miR-676-4386776	10.88442	1.09E-04	1.09E-04	1.175837	15.66903	23.43819	24.64041	13.40582	12.90395	flagged	valid	target_not_	-3.27654	Significant
rno-miR-20a#-4381116	13.17284	5.27E-05	5.27E-05	1.430671	18.91008	23.43819	24.64041	11.12784	10.60508	flagged	valid	target_not_	-3.96542	Significant
hsa-miR-29b-4373288	-4.88431	1.10E-02	1.10E-02	-2.18898	-4.5289	20.4429	17.80877	24.17213	23.84815	flagged	flagged	no_detecti	1.470322	Significant
mmu-miR-201-4373310	-3.16824	4.62E-03	4.62E-03	-1.34486	-5.80916	20.65599	21.02781	24.17213	23.84815	flagged	flagged	no_detecti	0.953735	Significant
mmu-miR-380-3p-4373350	4.596448	4.33E-03	4.33E-03	-1.28438	5.91586	23.43819	24.64041	19.9914	18.89431	flagged	flagged	no_detecti	-1.38367	Significant
mmu-miR-719-4386749	-6.47046	3.13E-03	3.13E-03	-0.99109	-6.47028	15.45814	15.52583	20.76265	23.16226	flagged	flagged	no_detecti	1.947804	Significant
rno-miR-421-4381122	9.664258	1.59E-04	1.59E-04	1.01755	14.2246	23.43819	24.64041	14.22727	14.52282	flagged	flagged	no_detecti	-2.90923	Significant
hsa-miR-200b-4381028	-10.0982	5.73E-03	5.73E-03	-1.54751	-5.46832	16.39139	11.43242	24.17213	23.84815	valid	flagged	calibrator_	3.039871	Significant
hsa-miR-34a-4373278	-1.8796	2.34E-02	2.34E-02	-2.95904	-3.60451	12.9849	12.9626	14.95602	14.75068	valid	flagged	calibrator_	0.565815	Significant

Supplement 2. Additional change of microRNA in aging Muscle.

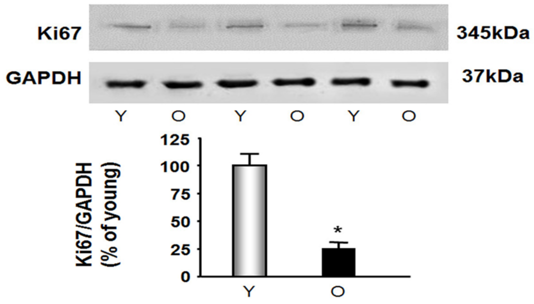

Supplement 3. Ki67 was decreased in aged mice muscle *vs* young's. The Ki67 expression, a marker of proliferation, was assessed by western blotting in muscle lysates from young and old mice. Results in the bar graph compare the densities of protein bands in aged muscle expressed as percentage chanfe from levels in young mice. All band densities were nirmilized to the density of GAPDH (Bars: mean ± s.e.; n=4; *p<0.05 *vs* young)

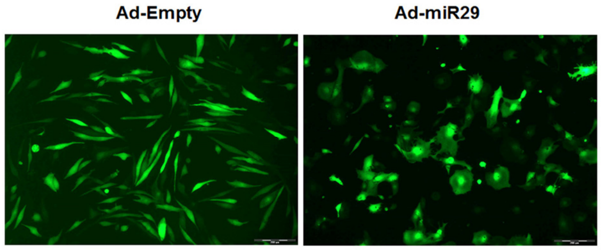

Supplement 4. miR29 changed cell shape in MPCs. MPCs supplied miR-29 exogenously led to flat cells resembling a fried egg, which indicated cell senescence.

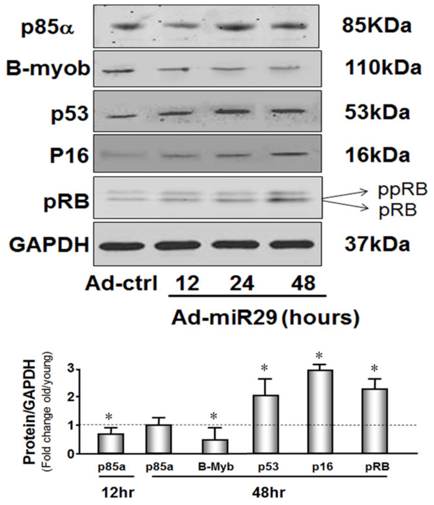

Supplement 5. miR29 increases cellular arrest in MPCs. The protein levels of B-myb, p53, $p16^{Ink4A}$, RB, and GAPDH were measured in MPCs treated with either control or Ad-miR-29. Two bands of the RB protein were detected. The lower band in hypophosphorylated RB (pRB; MW 112kDa). The bar graph shows the density of each 48 hour protein band expressed as a fold change from control levels (control set to 1 and indicated by horizontal line in the graph). All band densities were normalized using the density of GAPDH (Bars: mean ± s.e.; n=9; *p<0.05 *vs.* Ad-ctrl).

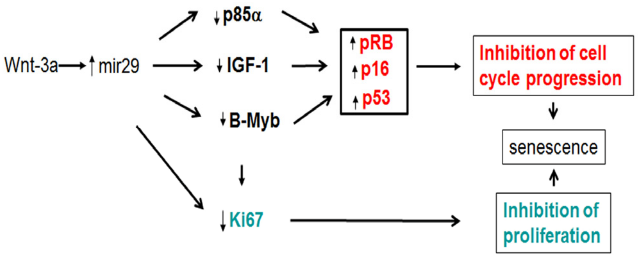

Supplement 6. The mechanism of miR29-induced senescence. With aging, the miR-29 promoter is activated by Wnt-3a. miR-29 then reduces levels of IGF-1, p85 and B-myb by directly binding to the 3'-UTR of each mRNA. Reduced levels of IGF-1, p85 and B-myb increase the level of cell arrest proteins (p53, p16 and RB). The increase in cell cycle arrest proteins block progression through the cell cycle and inhibit cell proliferation and induce cellular senescence.
